# Kilohertz Frequency Deep Brain Stimulation Is Ineffective at Regularizing the Firing of Model Thalamic Neurons

**DOI:** 10.3389/fncom.2016.00022

**Published:** 2016-03-15

**Authors:** João Couto, Warren M. Grill

**Affiliations:** ^1^Department of Biomedical Engineering, Duke UniversityDurham, NC, USA; ^2^Theoretical Neurobiology and Neuroengineering Laboratory, Department of Biomedical Engineering, University of AntwerpAntwerp, Belgium

**Keywords:** high frequency stimulation, computational model, informational lesion, conduction block, desynchronization

## Abstract

Deep brain stimulation (DBS) is an established therapy for movement disorders, including tremor, dystonia, and Parkinson's disease, but the mechanisms of action are not well understood. Symptom suppression by DBS typically requires stimulation frequencies ≥100 Hz, but when the frequency is increased above ~2 kHz, the effectiveness in tremor suppression declines (Benabid et al., 1991). We sought to test the hypothesis that the decline in efficacy at high frequencies is associated with desynchronization of the activity generated within a population of stimulated neurons. Regularization of neuronal firing is strongly correlated with tremor suppression by DBS, and desynchronization would disrupt the regularization of neuronal activity. We implemented computational models of CNS axons with either deterministic or stochastic membrane dynamics, and quantified the response of populations of model nerve fibers to extracellular stimulation at different frequencies and amplitudes. As stimulation frequency was increased from 2 to 80 Hz the regularity of neuronal firing increased (as assessed with direct estimates of entropy), in accord with the clinical effects on tremor of increasing stimulation frequency (Kuncel et al., 2006). Further, at frequencies between 80 and 500 Hz, increasing the stimulation amplitude (i.e., the proportion of neurons activated by the stimulus) increased the regularity of neuronal activity across the population, in accord with the clinical effects on tremor of stimulation amplitude (Kuncel et al., 2007). However, at stimulation frequencies above 1 kHz the regularity of neuronal firing declined due to irregular patterns of action potential generation and conduction block. The reductions in neuronal regularity that occurred at high frequencies paralleled the previously reported decline in tremor reduction and may be responsible for the loss of efficacy of DBS at very high frequencies. This analysis provides further support for the hypothesis that effective DBS masks the intrinsic patterns of activity in the stimulated neurons and replaces it with regularized firing.

## Introduction

Deep brain stimulation (DBS) is an effective treatment for the motor symptoms of movement disorders including essential tremor, Parkinson's disease, and dystonia, and recent data suggest that it may also be effective to treat epilepsy, obsessive-compulsive disorder, and depression. Although the mechanisms of action of DBS remain unclear, its effects are strongly dependent on stimulation frequency (Birdno and Grill, 2008). Symptom reduction is typically observed only when the stimulation frequency is ≥90 Hz, and lower frequencies are either ineffective or may exacerbate symptoms (Benabid et al., 1991; Ushe et al., 2004; Kuncel et al., 2006).

One hypothesis that accounts for the strong frequency-dependent effects of DBS, as well as the similarity of the overt effects of high frequency DBS and ablative lesion, is that DBS masks the intrinsic patterns of activity in the stimulated neurons and replaces it with regular firing (Grill et al., 2004). Empirical support for this hypothesis—termed the “information lesion”—comes from observations that: (1) DBS regularizes the firing patterns of downstream neurons (Hashimoto et al., 2003), (2) there is a strong correlation between the regularization of model neuron firing patterns and tremor reduction as a function of the frequency and amplitude of DBS (Kuncel et al., 2007), and (3) random patterns of stimulation, even when delivered at average frequencies of 130–185 Hz, are not effective at relieving symptoms (Birdno and Grill, 2008; Dorval et al., 2010). Further, a recent study demonstrated that DBS delivered at a symptom-relieving amplitude did indeed modulate and produce a partial lesion of information transmission in the basal ganglia (Agnesi et al., 2013). The objective of the present analysis was to test further the hypothesis that effective DBS regularizes neural firing by determining whether the observed loss of efficacy of DBS at kilohertz frequencies (Benabid et al., 1991) was the result of a loss of regularization of neuronal firing.

**Figure 1:**
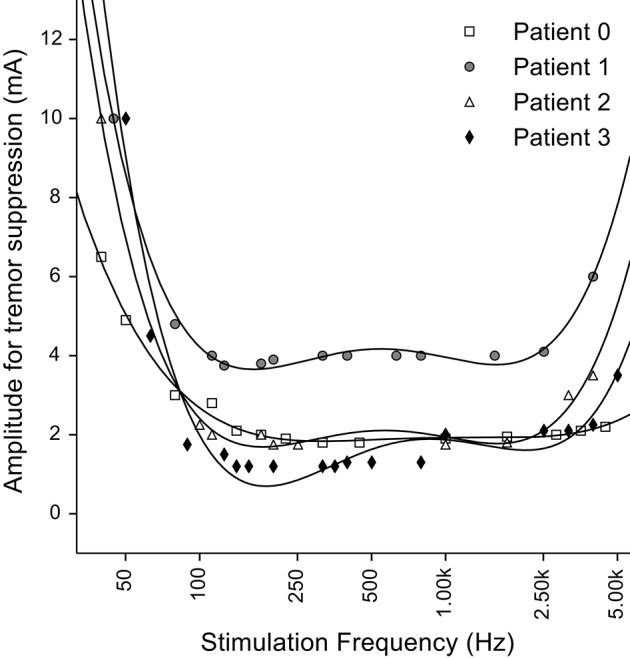
**Effect of stimulation frequency on the stimulation amplitude required for tremor suppression by thalamic deep brain stimulation (DBS)**. Fourth order polynomial functions were used to fit the experimental data (points) as in the original figure. Original data from Benabid et al. (1991).

In one of the earliest studies of thalamic DBS to treat tremor, Benabid and colleagues measured the threshold stimulation intensity required to suppress tremor as a function of the stimulation frequency (Benabid et al., 1991). The threshold for tremor suppression decreased monotonically as the frequency was increased from 50 to 150 Hz, and then remained constant up to frequencies of ~1 kHz (Figure 1), consistent with subsequent studies of the frequency dependence of symptom reduction (Birdno and Grill, 2008). However, when the stimulation frequency was increased above ~2 kHz, the stimulation intensity required to suppress tremor increased (i.e., the effectiveness of the DBS was decreased). One might expect that stimulation with pulses delivered during the refractory period simply leads to firing at factors of the stimulation rate, but this would not explain the loss of effectiveness. Instead, this finding is consistent with the regularization hypothesis if kHz frequency stimulation leads to irregular patterns of neuronal firing. Very high rate stimulation can lead to a variety of cellular responses (Weinberg, 2013), and the variability of neuronal responses increases in response to kHz frequency stimuli (Mino and Rubinstein, 2006). Rather than simply firing in response to every other or every nth stimulus pulse, the latencies of responses to stimuli within the relative refractory period become highly variable and neurons begin to fire in a pseudo-random pattern (Bowman and McNeal, 1986; Rubinstein et al., 1999).

The objective of the present analysis was to test the hypothesis that the observed loss of efficacy of DBS at kHz frequencies is the result of the onset of irregular and desynchronized firing across the population of stimulated neurons as the inter-pulse interval entered the neuronal refractory period. The results provide further support for the hypothesis that effective DBS masks the intrinsic patterns of activity in the stimulated neurons and replaces it with regularized firing (Grill et al., 2004).

## Materials and methods

We used a computational model of a mammalian myelinated axon to quantify the effects of high frequency stimulation on the firing of thalamic neurons, and analyzed the irregularity of neural firing using direct entropy estimation techniques. During extracellular stimulation of CNS neurons the axon is the site of action potential initiation (Nowak and Bullier, 1998; McIntyre and Grill, 1999) including during thalamic DBS (McIntyre et al., 2004) and tremor suppression is correlated with activation of thalamic axons (Holsheimer et al., 2000). Therefore, we used a model that only included a myelinated axon (Salami et al., 2003), rather than a full representation of the soma and dendrites of thalamic neurons. Further, previous computational studies of the response of cochlear neurons to high frequency stimulation showed that stochastic gating of channels had a strong effect on the threshold and latency of action potential generation (Rubinstein et al., 1999). Therefore, we extended the previous model to include stochastic representations of the ionic channels.

### Model axons

We use a multi-compartment cable model axon (5.7 μm in diameter) implemented in NEURON v7.2 (Hines and Carnevale, 1997) that included separate representations of the node, paranodal, and internodal regions, and a full complement of non-linear ionic conductances (available in ModelDB, ref: 3810). The model reproduced a wide range of experimental data (McIntyre and Grill, 2002), and was used in several studies of kHz frequency block of conduction (Bhadra and Kilgore, 2005; Bhadra et al., 2007). We include data in the Supplementary Material (Supplementary Figure 4) to support that our results also apply to other fiber diameters.

Both deterministic and stochastic versions of the fast sodium and slow potassium channels were implemented, while only a deterministic version was used for the persistent sodium channel. A Markov scheme was used to represent the gating dynamics of the ionic channels (Supplementary Figure 1) using the Channel Builder module of NEURON. The individual nodes contained varying densities of sodium and potassium channels, but the maximum conductances per node were kept constant across versions of the stochastic models and were similar to the deterministic model. The nodal membrane contains ~12,000 channels/μm^2^ (Ritchie and Rogart, 1977), and according to our nodal dimensions, this corresponded to ~71,000 channels per node. The density of potassium channels is lower and a linear function was used to calculate the number of potassium channels based on the number of sodium channels (N_K_ = N_Na_∗18/60) (Chow and White, 1996). Since the effect of stochastic ion channels is amplified for small number of channels (Rubinstein et al., 1999), we conducted simulations with 200–10,000 sodium channels per node to approximate low and high densities of channels, and compared the results to a deterministic model with similar parameters.

### Intrinsic activity

**Figure 2:**
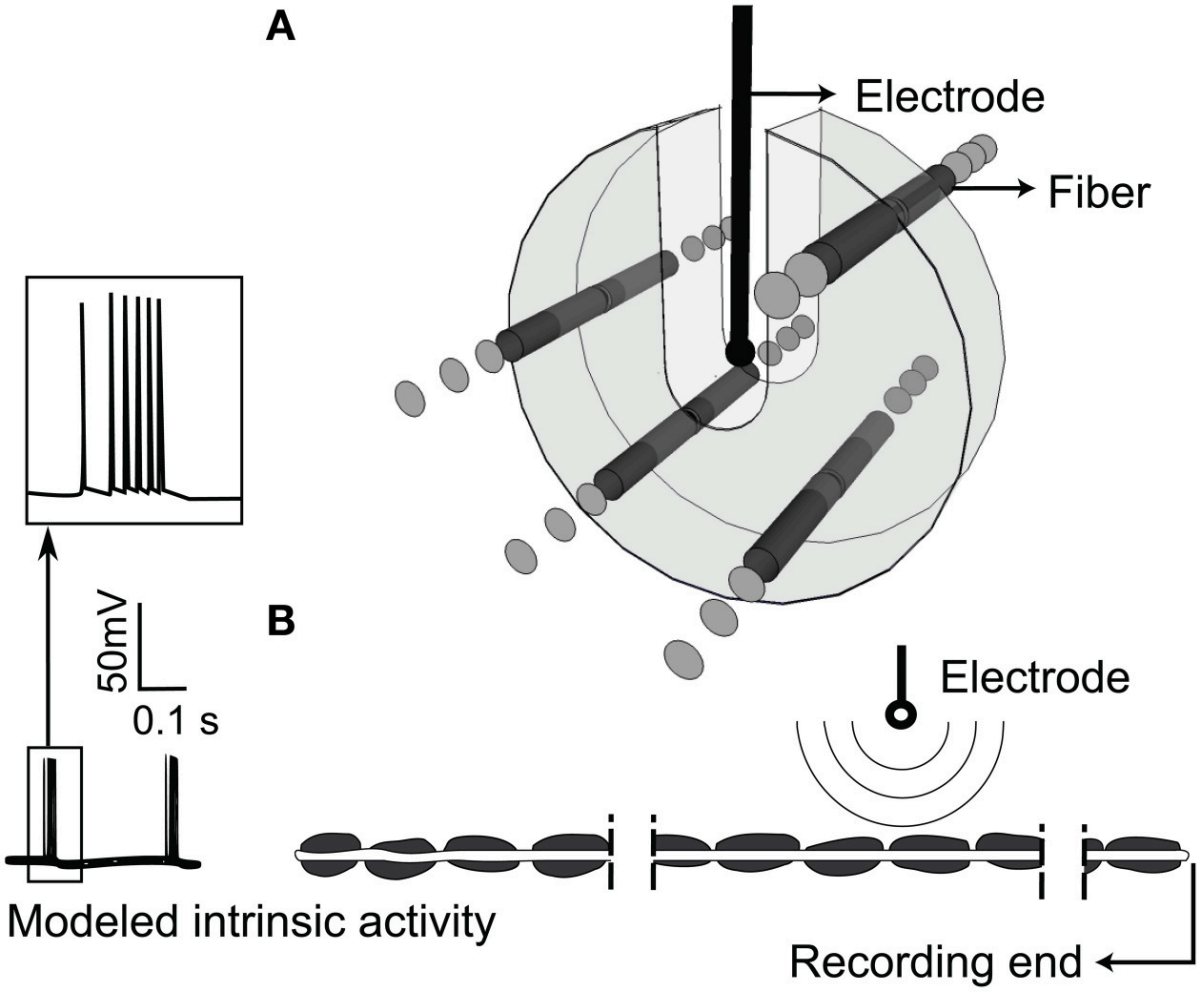
**Extracellular stimulation of model thalamic nerve fibers. (A)** Arrangement of the population of parallel model axons around the stimulating electrode. Fibers were not allowed in the U-shaped region around the electrode. **(B)** Intrinsic burst activity was evoked by intracellular stimulation at the proximal node of each fiber, the extracellular electrode was positioned closest to the 40th node from the distal end, and the transmembrane potential was recorded at the distal end.

The model neurons were given “intrinsic” patterns of activity by delivering patterned input via intracellular current injection to represent the bursting recorded in the thalamus of persons with Parkinson's disease (Magnin et al., 2000) or essential tremor (Hua et al., 1998; Hua and Lenz, 2005). The pattern of intrinsic activity had an inter-burst rate of 4.33 ± 0.06 Hz, and intra-burst rate of 243.40 ± 89.09 Hz (Magnin et al., 2000), and the timing of intracellular stimuli was generated using a highly realistic computational model of a thalamocortical relay neuron in bursting mode (McIntyre et al., 2004). The output of the model neuron was inserted by intracellular current injection at the proximal end of each axon (Figure 2B). Fibers with no modeled activity are termed “quiescent” and were modeled with 81 nodes (4 cm in length) to reduce computational time, while fibers with intrinsic bursting had 171 nodes (8.5 cm in length) to enable appropriate interaction between the intrinsic activity and the activity generated by simulated extracellular stimulation (see Supplementary Figure 2).

### Simulations

We simulated a population of 100 independent non-communicating parallel axons positioned randomly within 3 mm of a point source electrode in an infinite homogenous, isotropic medium with a resistivity of 500 Ω-cm (Zhang and Grill, 2010; Joucla et al., 2014; Figure 2A). Fibers were not allowed within a 0.635 mm radius sphere around the point source nor in the region above the source, space supposedly occupied by the DBS electrode. Under these conditions, the point source is a valid approximation of the DBS electrode (Zhang and Grill, 2010). The longitudinal position of the node nearest to the extracellular stimulating electrode was randomized within one half of one internodal length, and the electrode was located 40 nodes from the distal end of the axon. The transmembrane potential response to extracellular stimulation with trains of cathodic monophasic pulses, 90 μs in duration, was solved with backward Euler implicit integration and a 0.002 or 0.02 ms time-step. Stimulation frequency was varied from 2 to 9 kHz, and stimulation amplitude was varied from 30 to 200% of the current amplitude necessary to elicit action potentials in all axons at 100 Hz. In Supplementary Figure 6, we show data for stimulation with a sinusoidal waveform, and we observed results similar to those with monophasic pulses. With both waveforms we observed irregular firing and conduction block however, the relationship between conduction block threshold and signal frequency was inverted for cathodic monophasic pulses as compared to sinusoids (Supplementary Figure 3).

### Data analysis

We calculated the entropy of interspike interval (ISI) pairs (ISI_n_, ISI_n+1_) as a measure of the regularity of neuronal firing (Dorval, 2008; Dorval et al., 2008) according to

$$\begin{array}{l} H=-\overset{K}{\underset{k=1}{\sum }}P\left({pair}_{k}\right)log_{2}P\left({pair}_{k}\right) \end{array}$$

**Figure 3:**
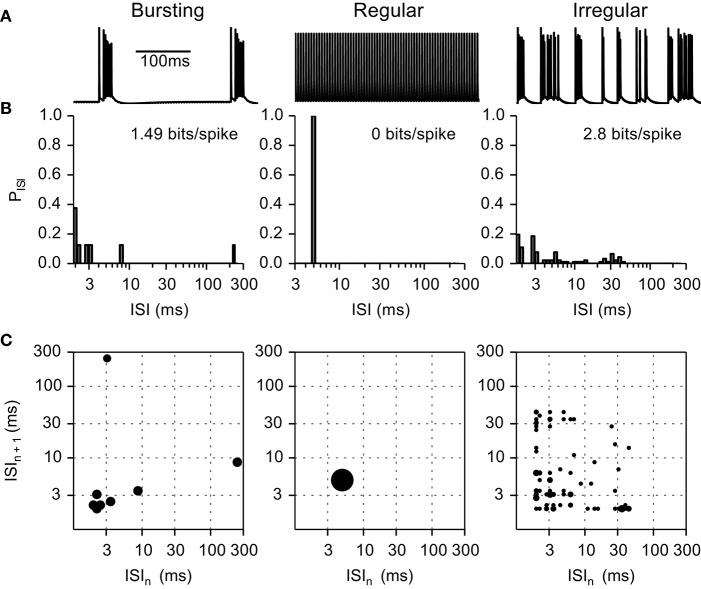
**Example of inter-spike interval (ISI) histograms and ISI pair histograms for fibers exhibiting intrinsic bursting activity, regular firing, and irregular firing. (A)** Transmembrane voltage in bursting, regular, and irregular firing model nerve fibers. **(B)** Histograms representing the probability of each ISI interval. **(C)** ISI pair maps illustrating the probability of an ISI pair. Larger circles represent higher probability. Bursting fibers (entropy 1.49 bits/spike) displayed peaks at short (intra-burst spikes) and at long (inter-burst spikes) ISIs; as compared to regular firing fibers (entropy 0 bits/spike) that displayed a single peak at the firing frequency in both the ISI histograms and the ISI pair histograms. Irregular fibers displayed very different firing patterns; in this example (entropy 2.8 bits/spike) the ISI histogram and ISI pair histograms are spread below 50 ms.

The probability distribution of ISIs were generated using logarithmic bins to remove the dependence of entropy on average firing rate (Dorval, 2008) with the bins determined by ${bin}_{k}={bin}_{0}\times 10^{\kappa ∕k}$, where *bin*_0_ = 0.4 *ms* (always shorter than the shortest ISI) and κ, the number of discrete bins per ISI decade, was set to 20 (Figure 3). The intrinsic activity of non-quiescent axons had firing pattern entropies of ~1.49 bits/spike, and we used a value 1.0 bit/spike as the cut-off between regular and irregular firing patterns.

**Figure 4:**
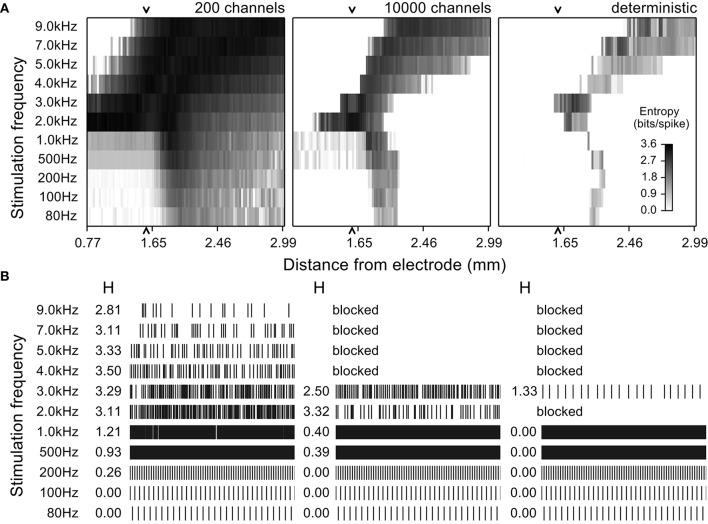
**Entropy maps and simulated spike trains from quiescent model nerve fibers for multiple stimulation frequencies and at different distances from the stimulating electrode. (A)** Entropy maps from stochastic model nerve fibers with 200 or 10,000 sodium channels per node and the deterministic model nerve fibers stimulated by an extracellular electrode at different frequencies. The amplitude of stimulation was set to activate 50% of the fibers at 100 Hz. The grayscale indicates the entropy of the spike train for a fiber at a particular distance. **(B)** Firing patterns (rastergrams) and entropy values (H) of an example model nerve fiber 1.59 mm from the electrode (arrow in the entropy maps) for each of the three models (three columns) across stimulation frequencies (rows). The low channel density stochastic model fired spontaneously with high entropy even in the absence of stimulation, but this was not observed in the high channel density stochastic model or in the deterministic model. In the later two cases, stimulation had similar effects for all frequencies.

**Figure 5:**
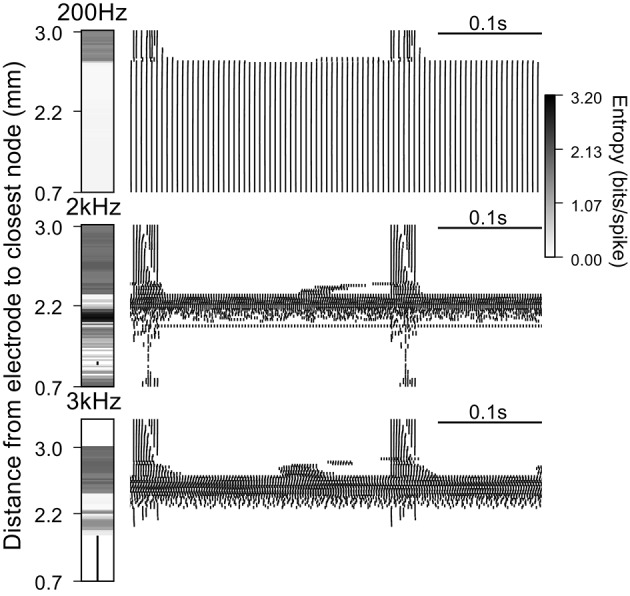
**Firing patterns generated by extracellular stimulation at 200 Hz, 2 kHz, and 3 kHz**. Raster plots and entropy maps (vertical bars indicate fibers where conduction was blocked) for a population of 100 deterministic model nerve fibers and a stimulation current of 1.6 mA, which evoked firing in ~75% of the fibers at 100 Hz.

We used comparatively short 1 s simulation epochs to reduce the overall computational demands. To estimate the effect of this choice on the entropy measurements, we simulated five fibers for 20 s during stimulation at 200, 2000, and 4000 Hz, and compared the estimates of firing pattern entropy to those from the 1 s simulations (Supplementary Table 1). The mean absolute error was only 0.19 bits/spike (compared to the range of entropies of 0–3.2 bits/spike in Figures 4, 5) indicating that the short simulation epochs were sufficient for assessing changes in entropy due to stimulation at different frequencies.

## Results

We first used computer simulations of quiescent fibers to show that for a large number of simulated channels, stochastic and deterministic models responded in a similar manner to high frequency stimulation. We then introduced an intrinsic pattern of irregular burst activity in model deterministic fibers and quantified the fraction of fibers that fired regularly at different stimulation frequencies. While activity was regularized by extracellular stimulation at frequencies between 100 and 1500 Hz, higher frequencies generated irregular firing patterns. Further increasing the stimulation amplitude or frequency blocked action potential conduction and increased the regularity of model neuron firing.

### Influence of stochastic channels on firing pattern entropy

We used quiescent stochastic and deterministic models to determine the effects of the stochastic properties of ion channels on the response of model nerve fibers to stimulation with frequencies between 80 and 9 kHz. Responses were dependent on stimulation frequency, the electrode to fiber distance, or, at a fixed distance, dependent on the stimulation intensity. The responses of the stochastic model with a high density of sodium channels (10,000 channels per node) approximated those of a deterministic model, albeit with a higher overall entropy. Conversely, the responses of the stochastic model with a low density of sodium channels (200 channels per node) were substantially more variable than those of the deterministic model, and, unlike the other two models, this low density stochastic model nerve fiber exhibited spontaneous firing.

The evoked patterns of activity in each model variant were quantified by calculating the firing pattern entropy for each axon in the population with the extracellular stimulation intensity selected to evoke action potential firing in half of the model axons at a stimulation frequency of 100 Hz (Figure 4). At frequencies below 1 kHz, fibers close to the electrode (< ~2 mm; bottom left quadrant in each map in Figure 4A) were activated at the stimulation frequency and fired in an entirely regular pattern that had firing pattern entropy of zero. Fibers at an intermediate distance responded with an irregular pattern of firing, as the stimuli were close to threshold intensity for these fibers, and the variability in firing pattern (entropy) was somewhat higher in the stochastic models than in the deterministic model. Stimulation at any frequency had little effect on axons that were distant from the electrode; these axons remained quiescent and had entropies of zero (lower right quadrant of the entropy maps in Figure 4A) in the 10,000 channel and the deterministic variants, while in the low channel density stochastic model, spontaneous action potentials produced non-zero entropy in the more distant fibers (Strassberg and DeFelice, 1993).

Higher stimulation frequencies produced block of conduction in fibers close to the electrode and the resulting cessation of activity produced firing pattern entropy of zero (upper left quadrant of the entropy maps in Figure 4A, for all model variants). The relationship between conduction block threshold and signal frequency was dependent on the signal waveform. Sinusoidal waveforms exhibited conduction block thresholds that increased with frequency (Supplementary Figure 3), while for cathodic monophasic waveforms the thresholds to produce either excitation or conduction block decreased as frequency was increased above 1 kHz. Thus, as we increased the frequency, the distance over which conduction block was generated increased, and the band of distances over which axons were activated in an irregular pattern shifted to distances farther from the electrode.

This analysis showed a remarkable similarity in the responses evoked across stimulation frequencies and distances in the high-channel-density stochastic model and the deterministic model. Although the absolute entropy was consistently higher in the high-channel-density stochastic model (mean absolute difference was 0.61 bits/spike), the patterns and changes in entropy were similar to those in the deterministic model, and to reduce computational cost and complexity we used the deterministic model to analyze the effects of DBS on intrinsically active (bursting) model nerve fibers.

### Effect of DBS frequency on firing pattern entropy

We used a population of 100 deterministic model nerve fibers, with intrinsic burst activity generated by intracellular current injection at one end of each fiber, to quantify the effect of extracellular stimulation on firing patterns. The ability to mask the intrinsic bursting in the model depends on the distance between the node closest to the electrode and the proximal end of the axon where the patterned intracellular stimuli were delivered (i.e., the length of the axon, Supplementary Figure 2). Example rastergrams in Figure 5 show the output firing of the population during extracellular stimulation at 200 Hz, 2 kHz, and 3 kHz. At 200 Hz, the closest ~75% of the fibers followed the stimulation train one for one, and had firing pattern entropy of zero. The remaining 25% of fibers continued to fire with the intrinsic burst activity, and stimulation-generated irregular activity was superposed on the intrinsic activity with latencies dependent on the electrode to fiber distance. Thus, these fibers had firing pattern entropies higher than the entropy of the intrinsic burst activity (1.49 bits/spike).

At 2 kHz, the fibers closest to the electrode exhibited partial conduction block. Some nodes of these fibers were very close to activation threshold and the action potentials recorded in the distal node were not due to direct propagation of the action potentials from the proximal node, but rather due to the activation of these intermediate nodes that was facilitated during the intrinsic bursts. The axons at intermediate distance from the electrode were excited by the stimulus, the intrinsic bursting activity was masked, and the regularized patterns of firing had comparatively low entropies. At positions farther from the electrode, fibers continued to fire with intrinsic burst activity and 2 kHz stimulation generated irregular activity with variable latency, resulting in higher firing pattern entropies. Thus, when the stimulation frequency was increased from 200 Hz to 2 kHz, DBS was less effective at masking the pathological bursting activity, generated less orderly firing, and the firing pattern entropy increased.

At 3 kHz, fibers close to the electrode were completely blocked for the same stimulation amplitude used above. Again, at intermediate distances, fibers exhibited low entropy patterns of firing due to excitation by the high frequency stimulation, and at farther distances fibers continued to fire with the intrinsic burst activity, as well as irregular stimulation generated activity, whose latency was dependent on the electrode to fiber distance. Thus, when the frequency was further increased, the overall entropy decreased due to the emergence of high frequency conduction block and, in some cases, more ordered, regular firing in response to the stimulation (see below).

**Figure 6:**
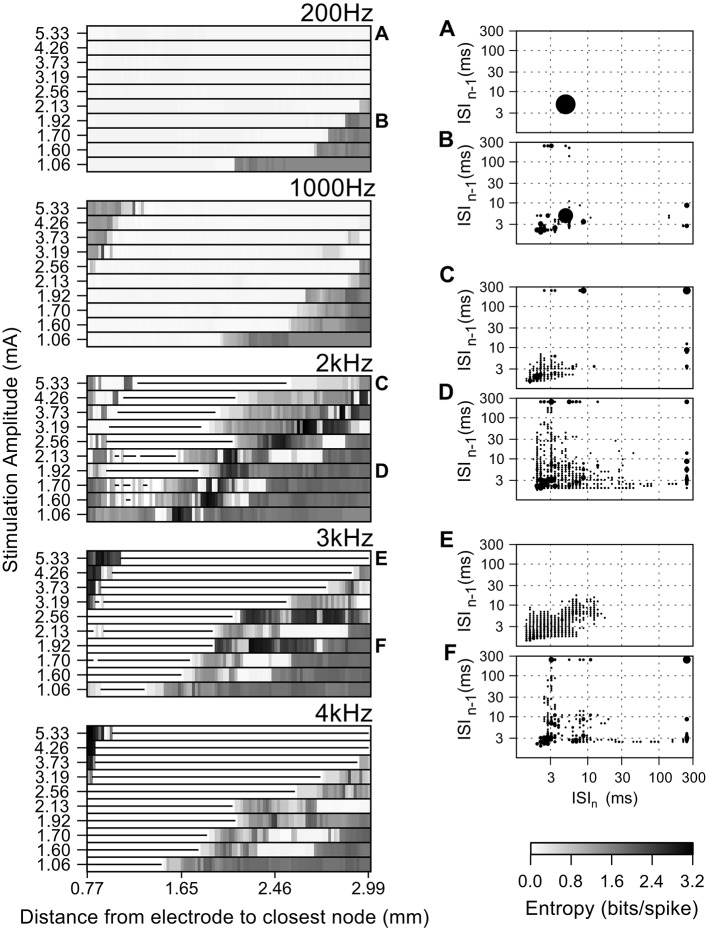
**Effect of the amplitude of the stimulating current on the firing pattern of model axons during high frequency extracellular stimulation**. Entropy maps for multiple frequencies and current amplitudes (left), and population ISI pair histograms (right), i.e., from all fibers at a particular current amplitude. The area of each circle represents the probability of a given ISI being followed by another. The ISI pair histograms **(A–F)** correspond to the labels in the left panels and refer to: **(A)** The intrinsic activity was masked by extracellular stimulation in all fibers and replaced by entirely regular firing, thereby reducing the ISI pair histogram to a single circle. **(B)** Stimulation regularized firing in 50% of the fibers, but did not mask the intrinsic activity in the remaining fibers. **(C–F)** Irregular firing patterns evoked by high frequency stimulation. Stimulation amplitudes higher than 3.19 mA at frequencies above 1 kHz generated irregular activity patterns in the fibers closer to the electrode due to activation initiating at nodes further away from the electrode. In the entropy maps, the horizontal lines indicate fibers where the action potential conduction was blocked by extracellular stimulation.

We quantified the effect of increasing stimulation amplitude on the entropy of the firing patterns of intrinsically bursting model nerve fibers at different electrode to fiber distances (Figure 6). At 200 Hz the stimulus regularized the firing in fibers closer to the electrode, and increasing the stimulation amplitude further increased the fraction of regularly firing fibers. At 1 kHz, the effects paralleled those of stimulating at 200 Hz; however, for higher stimulation amplitudes (above 3.19 mA), the activation of multiple neighboring nodes of Ranvier caused an increase in firing pattern entropy in fibers close to the electrode. Higher stimulation frequencies generated irregular patterns of activation in combination with block of conduction as seen in the panels for 2, 3, and 4 kHz.

The firing patterns of the fiber populations are summarized in the histograms of inter-spike interval pairs (the insets of Figure 6). The histograms during 200 Hz stimulation (Figures 6A,B) exhibited a large circle centered at (5 ms, 5 ms), reflecting the primary firing pattern at the stimulation frequency, and small, widely distributed circles reflecting the bursting and irregular firing in the more distant fibers at smaller stimulation amplitudes. The ISI pair histograms for stimulation at higher frequencies exhibited irregular patterns of firing as seen from the dispersed positions of more and smaller circles (Figures 6C,D). The circles on the histogram for 3 kHz (Figures 6E,F) have lower total areas than, e.g., at 2 kHz, because some fibers underwent conduction block and thus did not contribute ISI pairs.

### Conduction block at high stimulation frequencies

**Figure 7:**
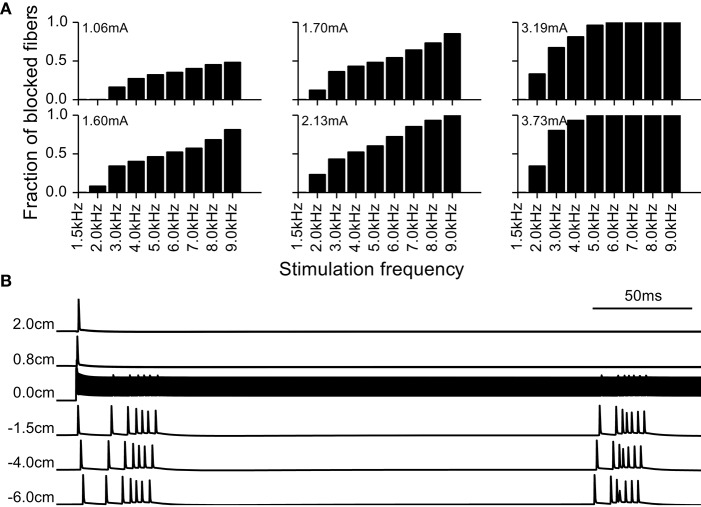
**Block of action potential conduction by high frequency stimulation. (A)** Fraction of model nerve fibers undergoing conduction block at different stimulation frequencies and amplitudes. **(B)** Example of a model nerve fiber undergoing conduction block; node at 0 cm is the closest to the extracellular electrode and −6 cm is the location of the node where the intrinsic burst activity was introduced with an intracellular electrode. The intrinsic activity does not propagate through the central node at 0.0 cm, immediately beneath the stimulating electrode.

We observed that as the amplitude and frequency of stimulation were increased, fibers exhibited conduction block and failed to propagate the intrinsic burst activity. After an initial transient response at the onset of the kHz frequency signal, stimulation generated very little activity in the blocked fibers. As there is previous evidence for DBS producing a cessation of activity in intrinsically active neurons, and such a cessation of activity can have a profound effect on firing pattern entropy, we analyzed the conduction block in more detail (Figure 7). At the amplitudes that resulted in conduction block, the onset of the kHz-frequency extracellular stimulus typically generated at least one action potential that propagated in both directions in the model fibers (see bottom panel of Figure 7, first action potential initiates at node closest to electrode (0.0 cm) and propagates in both directions). This onset response was followed by ongoing high frequency oscillations in transmembrane potential superimposed on tonic depolarization, which resulted in conduction block of the action potentials constituting the intrinsic burst activity generated by intracellular current injection at the proximal end of the model fiber (Figure 7, bottom panel). The fraction of fibers in the population that exhibited conduction block of intrinsic burst activity increased with the frequency and amplitude of stimulation (Figure 7, top panel).

### Effect of DBS frequency

**Figure 8:**
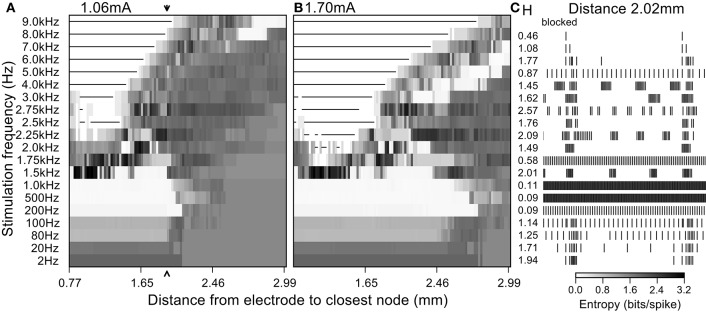
**Firing patterns generated by extracellular stimulation at multiple frequencies**. Stimulation at a current sufficient to evoke firing in **(A)** 50% and **(B)** 80% of the fibers at 100 Hz. **(C)** 400 ms of the simulated spike trains for a single model nerve fiber located 2.02 mm away from the electrode (arrow in left entropy map) and the entropy values obtained when stimulating at each frequency.

Fibers stimulated by DBS below 1 kHz tended to follow the frequency of the stimulus dependent on the amplitude and/or the distance to the electrode, and this was manifested in the firing pattern entropy in different ways. At 2–80 Hz (Figure 8) the entropy of the stimulated fibers was higher than that of the non-stimulated fibers because there was a superposition of the intrinsic burst activity and new stimulus-generated activity. Stimulation frequencies between 80 Hz and 1 kHz masked the intrinsic burst activity and established regular firing in the population of stimulated fibers, thereby reducing entropy to zero. As expected, increasing the amplitude of the stimulus resulted in an increase of the number of fibers firing regularly (i.e., the distance over which fibers fired regularly).

At frequencies higher than 1 kHz, irregular patterns of activity and conduction block dominated the response of the population despite the regularity of the stimulus (Figure 8). The appearance of irregular firing patterns (and hence the increased entropy generated with stimulation frequencies above 1 kHz) was attributable mainly to two phenomena: (1) direct extracellular activation, as was responsible for highly irregular firing patterns as seen in quiescent fibers (Figure 4), and (2) partial conduction block that failed to prevent completely propagation of the intrinsic burst activity (as seen in Figure 8 for stimulation frequencies of 2 kHz and 3 kHz).

### Effect of DBS amplitude

**Figure 9:**
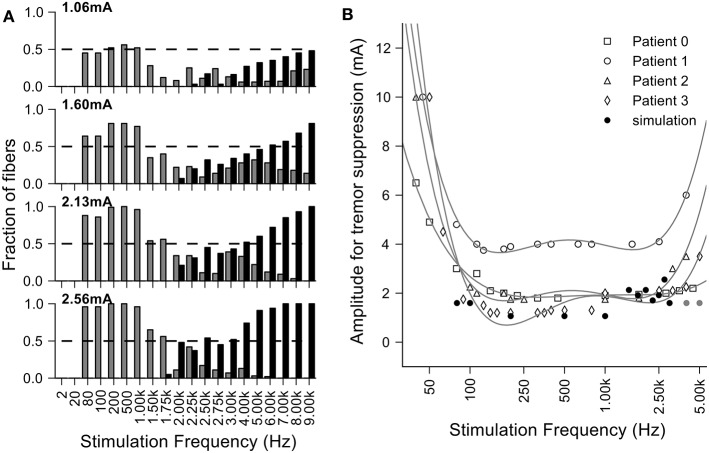
**Loss of efficacy of DBS at very high frequencies can be explained by increases in entropy. (A)** Fraction of fibers where the entropy of the output firing is <1 bit per spike at different amplitudes and frequencies of stimulation. The fibers that have a regular output pattern are depicted in gray and those that undergo conduction block in black. For large stimulation currents and higher frequencies, we did not see regularized firing due to the occurrence of conduction block and the finite extent of the population of model nerve fibers. **(B)** Overlay of the clinical data from Benabid et al. (1991) (each point was normalized by the per patient minimum value and then multiplied by the average of the minimum values across patients) and the minimal amplitude to regularize half of the fibers in the simulations.

Increasing the amplitude of extracellular stimulation had strikingly different effects on neuronal activity and entropy, dependent on the stimulation frequency. In the case of DBS at 200 Hz (Figure 6) increasing the amplitude reduced the spike train entropy of more fibers in the population by replacing the intrinsic burst activity with regular firing time-locked to the stimulation. Similar effects were observed for stimulation frequencies up to 1 kHz, but for higher frequencies, increasing the amplitude decreased the overall entropy due to an increase in the proportion of fibers undergoing conduction block and an increase in the proportion of fibers firing regularly (Figure 6). In Figure 9, we used an entropy threshold to classify the firing patterns of each fiber as regular (<1 bit/spike, excluding fibers undergoing conduction block) or irregular; there was an increase in the number of fibers with regular firing patterns for frequencies above 1 kHz when the stimulation amplitude was increased.

## Discussion

We assessed the effects of high frequency stimulation on populations of simulated axons using model fibers incorporating stochastic or deterministic representations of voltage-gated ion channels. In light of recent studies that correlated firing pattern regularity with DBS efficacy (Grill et al., 2004; Dorval et al., 2008), we computed the entropy of the firing pattern of each fiber when stimulated at different frequencies and amplitudes. At stimulation frequencies above 2 kHz, we observed highly irregular firing patterns as a result of variable activation and silencing of fibers close to the electrode (Figure 4). Similar irregular patterns of firing in response to high frequency stimulation have been observed in cochlear neurons (Litvak et al., 2001) and alpha motoneuron axons in the cat sciatic nerve (Bowman and McNeal, 1986) in response to kHz frequency stimulation. The effects we observed are consistent with the hypothesis that the observed loss of efficacy of DBS at frequencies >1 kHz (Benabid et al., 1991; Figure 1) is related to the inability the stimulation to regularize neuronal firing.

### Loss of efficacy of DBS at very high frequencies can be explained by increases in entropy

The generation of irregular patterns of activity in fibers during kHz frequency stimulation, and the subsequent emergence of regular patterns or conduction block when the current amplitude was increased, can explain the loss of efficacy with kHz frequency DBS reported in Benabid et al. (1991). The effects of stimulation amplitude on the firing of model fibers (Figure 9) can be related to the data from Benabid et al. (1991) if one selects a proportion of fibers (e.g., 50%) that are required to be firing regularly (i.e., entropy <1 bit/spike) as a threshold for effectiveness (dashed lines in Figure 9A). For frequencies between 80 Hz and 1 kHz, the stimulation amplitude to achieve regularization of 50% of the population was relatively constant. However, stimulation frequencies above 1 kHz required that the amplitude of stimulation be increased, as observed in the experimental data (Figure 9B). The changes in information content (entropy) of the model population paralleled the stimulation frequency dependent changes in stimulation amplitude necessary for tremor suppression, and this supports the hypothesis that DBS acts by masking intrinsic patterns of activity and replacing them with regularized patterns of neural firing.

### Stochastic representations of ion channels were not required for high frequency induced variability

We conducted initial simulations to determine the contributions of the stochastic properties of the ion channels to the firing of axons subject to high frequency stimulation. We compared simulations with quiescent stochastic model fibers containing variable numbers of channels, but keeping the nodal conductance constant to enable comparisons across channel densities and with the deterministic model. Stochastic fibers with high channel density exhibited firing patterns that strongly resembled those of the deterministic model under similar conditions, in agreement with previous computational studies (Strassberg and DeFelice, 1993; Chow and White, 1996). This result suggests that the stochastic properties of ion channels are not necessary for generating the irregular firing. Although low channel densities amplify stochastic effects (White and Rubinstein, 2000), there is a high density of channels present in the nodal membrane and the single channel conductances are relatively small (~10 pS; Ritchie and Rogart, 1977).

Extracellular stimulation at kHz frequencies generated irregular firing patterns in deterministic model nerve fibers, indicating not only that this phenomenon does not require stochastic properties of the ionic channels but also that it is not due to interactions with the intrinsic activity pattern. For frequencies between 4 and 10 kHz further increasing the stimulation amplitude suppressed the irregular firing patterns by generating conduction block. Conduction block was significantly harder to achieve in fibers close to the electrode with frequencies between 2 and 3 kHz, and model neurons showed irregular patterns at high amplitudes. Although we did not examine this phenomenon exhaustively, shifts in the site of activation to different nodes along the length of fiber appeared to be responsible for the observed irregular patterns of activity.

### High frequency conduction block and firing pattern irregularity generated by kHz frequency DBS

In our simulations we observed that depending on the stimulation parameters and the fiber location, kHz frequency electrical stimulation could (1) generate conduction block of activity in the model nerve fibers or (2) generate highly irregular patterns of activity in the model nerve fibers. Conduction block with kHz frequency signals has been demonstrated experimentally (Bowman and McNeal, 1986; Joseph and Butera, 2011) and in simulations (Kilgore and Bhadra, 2004; Bhadra and Kilgore, 2005; Bhadra et al., 2007). We observed that the proportion of fibers that experienced conduction block depended on the frequency and amplitude of the stimulus (Figure 7). The threshold for conduction block using sinusoidal and squarewave stimuli increases proportional to the frequency of the stimulus (Bhadra et al., 2007). However, with monophasic cathodic stimuli we observed that this relationship was reversed in relation to sinusoidal or squarewave stimulation under the same conditions (Supplementary Figure 3). Further, prior studies (Bhadra and Kilgore, 2005; Bhadra et al., 2007) showed that the threshold for conduction block with kHz frequency stimulation was higher for smaller diameter fibers, which raised the question of whether the choice of fiber diameter perhaps biased our results. Therefore, we conducted simulations with 10 and 15 μm fibers and obtained results similar to those with 5.7 μm diameter fibers (Supplementary Figure 4).

DBS at clinically-effective frequencies entrains spike activity both downstream and upstream from the site of stimulation, although the entrainment occurs only for a fraction of the stimulation pulses (Agnesi et al., 2015). Changes in entrainment with stimulation frequency may contribute to increasing the number of irregular firing neurons; however, our study suggests that the irregularity induced at the stimulation site alone can explain the increase in threshold for tremor suppression. In addition, HFS may cause depletion of neurotransmitter resulting in attenuation of cortical responses to thalamic stimulation (Bellinger et al., 2008). Irregular, lower firing rate activity patterns—such as those generated by kHz frequency HFS—may not result in depletion of neurotransmitter pools and may therefore be transmitted downstream and thereby contribute to the increase in threshold for tremor suppression.

### Model limitations

This modeling study enabled the identification of possible causes of the increase in stimulation amplitude required to suppress tremor with kHz frequency DBS. However, the model included a limited representation of possible geometries; the fibers were all orientated parallel to each other and had a linear geometry, which is not representative of the true anatomical arrangement of axons around the electrode. Furthermore, the model did not include other neuronal elements that are also affected by extracellular stimulation and may contribute to effects of thalamic DBS on tremor. Accumulation of potassium within the peri-axonal space may contribute to functional block of axons in response to high-frequency stimulation (Bellinger et al., 2008), and our model did not account for effects of potassium accumulation.

## Conclusion

Increased irregularity in the pattern of neural firing has been suggested as the cause of symptom exacerbation by low frequency DBS while masking of intrinsic neural activity and the consequent regularization of neuronal firing by high frequency DBS has been hypothesized as responsible for the therapeutic success of DBS (Grill et al., 2004). Using computer simulations, we showed that irregular and desynchronized patterns of activation can appear in response to DBS at frequencies >1 kHz, and these irregular patterns of neural activity may account for the loss of efficacy reported in Benabid et al. (1991).

The loss of tremor suppression by thalamic DBS at kHz frequencies can be explained by the increases in the entropy of neuronal firing generated by kHz frequency DBS in model neurons. Entropy was subsequently reduced when the amplitude of the stimulus was further increased, consistent with the experimental observations of increased stimulation amplitude thresholds for tremor reduction with kHz frequency DBS (Benabid et al., 1991). The parallels between the stimulation frequency- and amplitude-dependent changes in firing pattern entropy in model neurons and the changes in thresholds to achieve tremor reduction by thalamic DBS provide further support for the hypothesis that effective DBS masks the intrinsic patterns of activity in the stimulated neurons and replaces it with regular activity patterns (Grill et al., 2004).

## Author contributions

JC performed computer simulations and analyzed the data. JC and WG designed the research and wrote the paper.

## Funding

This work was supported by NIH R01 NS040894 and R37 NS040894.

### Conflict of interest statement

The authors declare that the research was conducted in the absence of any commercial or financial relationships that could be construed as a potential conflict of interest.
