# Supplementary material for: Kilohertz Frequency Deep Brain Stimulation Is Ineffective at Regularizing the Firing of Model Thalamic Neurons
Source: Front Comput Neurosci. 2016 Mar 15;10:22. doi: 10.3389/fncom.2016.00022 (PMC4791372; doi:10.3389/fncom.2016.00022)
Supplement: Supplementary file 1 [file Image1.pdf]

Supplementary Material

Kilohertz frequency deep brain stimulation is ineffective at regularizing the firing of model thalamic neurons

João Couto, Warren M. Grill*

*** Correspondence:** Warren M. Grill: warren.grill@duke.edu

1 Supplementary Figures and Tables

1.1 Supplementary Figures

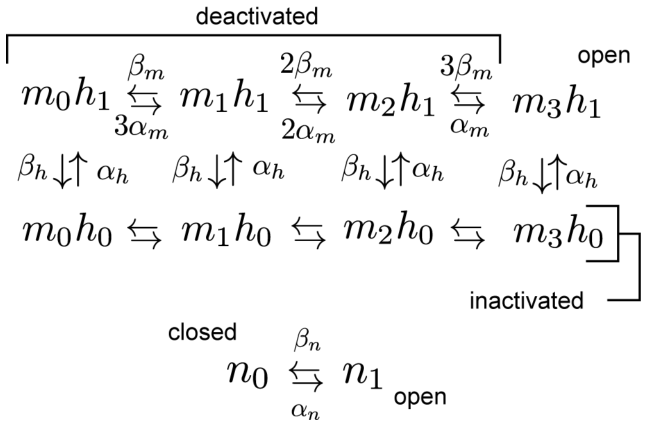

Supplementary Figure 1. Kinetic schemes used to describe the gating variables of sodium and potassium.

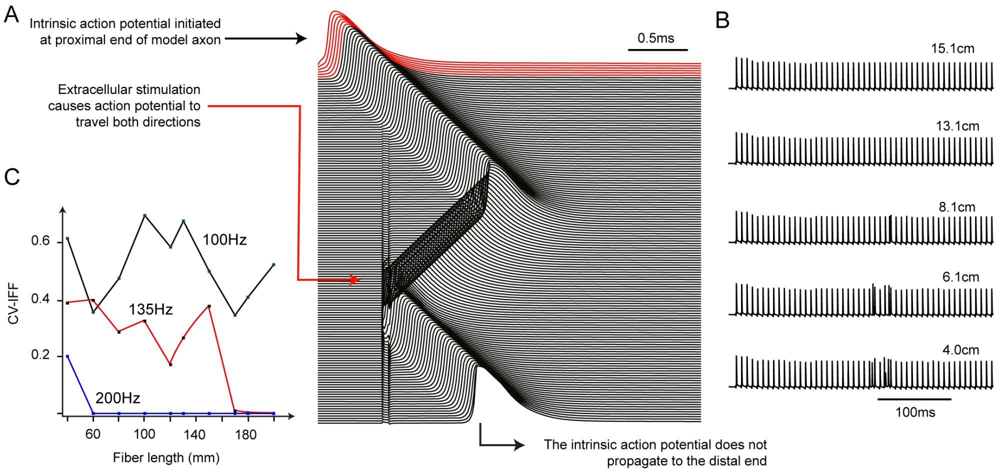

Supplementary Figure 2. Ability to suppress action potentials depends on the length of the model fiber. Using output regularity as a measure of DBS efficacy we conducted simulations with different axonal lengths and measured the efficacy of high frequency stimulation at frequencies used in the clinic. We observed that the intrinsic action potential generated by intracellular stimulation at the proximal end of the model axon was suppressed completely at 135 Hz only when fibers were longer than 170 mm with the extracellular electrode placed 40 nodes away from the ends of the axon in all simulations. (A**)** Example of modeled intrinsic action potential propagation and suppression by high frequency stimulation; traces are the membrane voltage measured in each node of Ranvier. (B) Transmembrane voltage for model axons of different lengths stimulated at the same frequency. (C) Regularity of neuronal firing measured as the coefficient of variation of the instantaneous firing frequency versus the length of the model axon measured for multiple frequencies. 100 Hz was not sufficient to mask the intrinsic action potential at any length.

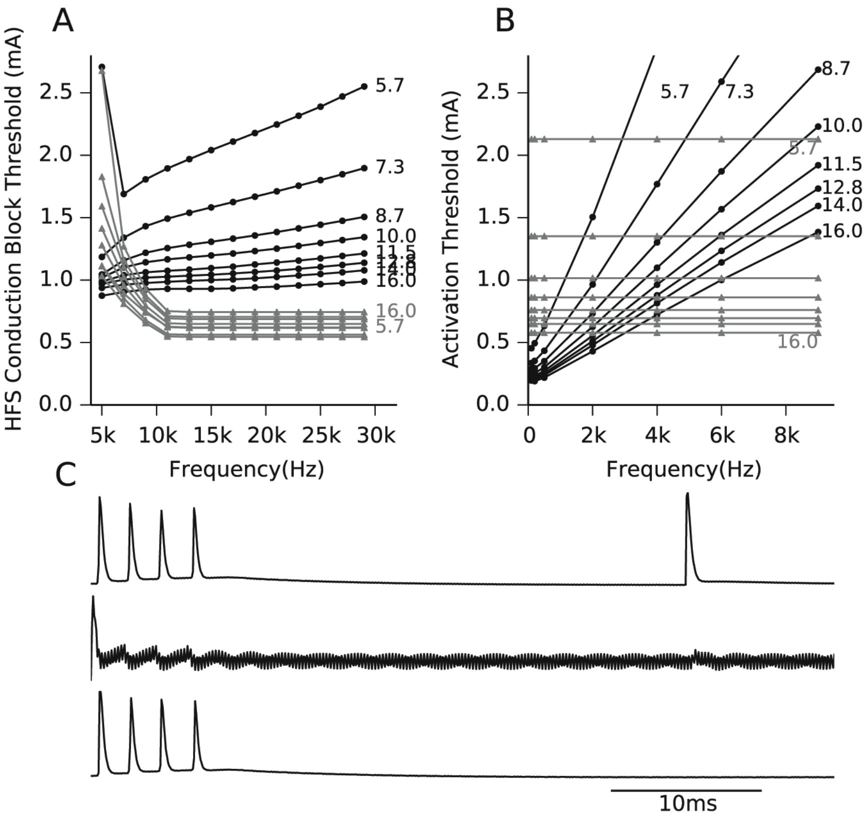

Supplementary Figure 3. Conduction block and activation threshold – as a function of extracellular stimulation frequency – for model axons with different diameters (µm). (A) Conduction block for fiber 1mm from the electrode. Black circles are for a sinusoidal stimulation waveform, as used in Bhadra et al. 2007, and gray triangles for a cathodic square monophasic wave with a pulse width of 90 µs. (B) Activation threshold for extracellular stimulation of a fiber at 3 mm using a single stimulation cycle. (C) Voltage recorded during one of the simulations in A for (from top to bottom) nodes 0, 25 and 50 where node 0 is where the intracellular stimulation is delivered, node 25 is closest to the stimulating electrode and node 50 the distal end of the model axon where the output was recorded. Example is a 10 µm axon stimulated at 9 kHz and 1.14mA at an electrode to axon distance of 1 mm.

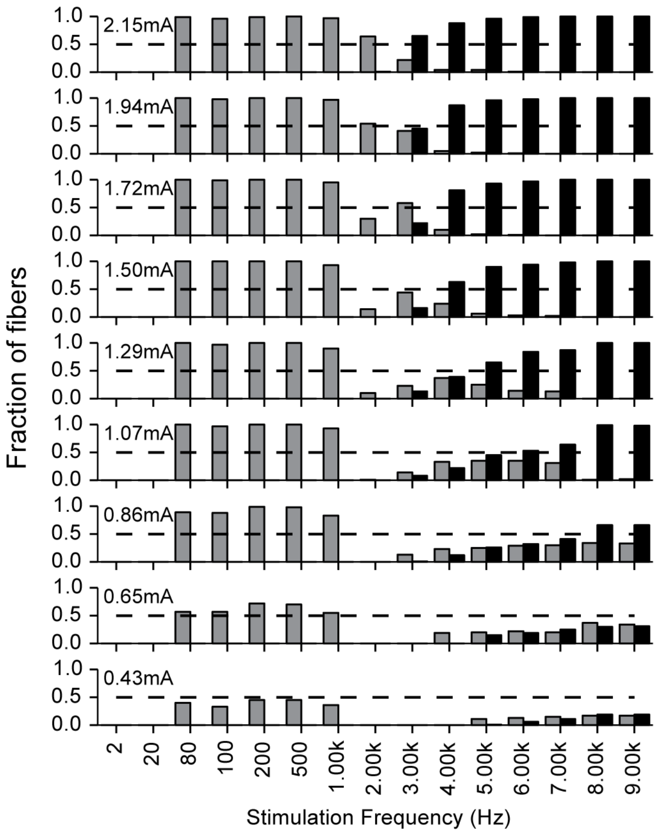

Supplementary Figure 4. Summary plots for populations of 10 μm diameter fibers (100 axons with 81 nodes of Ranvier and intrinsic activity) as in Figure 9 of the manuscript. Bar plots of fraction of fibers undergoing conduction block (black) and fibers with regular output (gray).

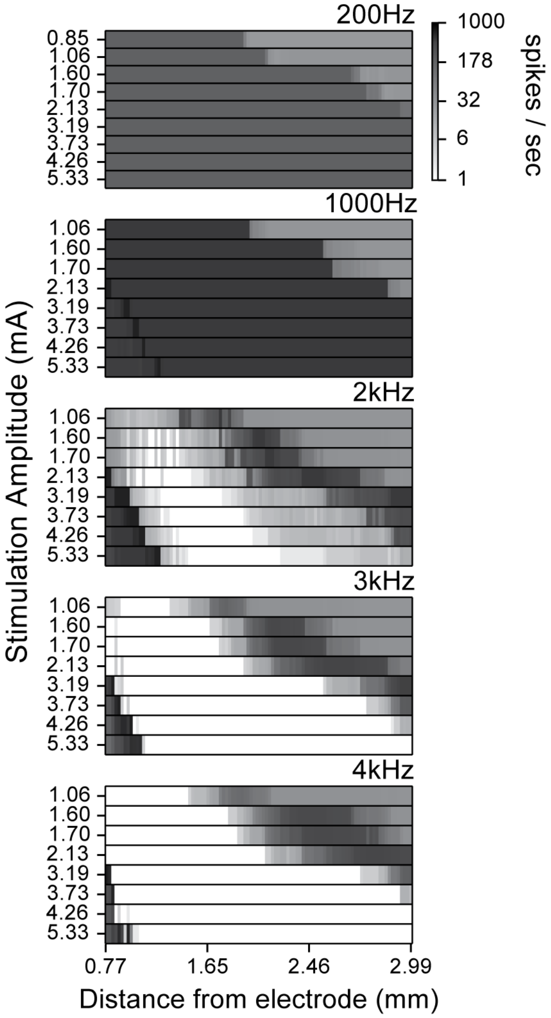

Supplementary Figure 5. Firing rates in response to extracellular stimulation at different frequencies and amplitudes in a model with intrinsic activity. Each row represents the firing rate of 100 fibers in response to extracellular stimulation at a particular frequency. The fibers are sorted by proximity to the electrode.

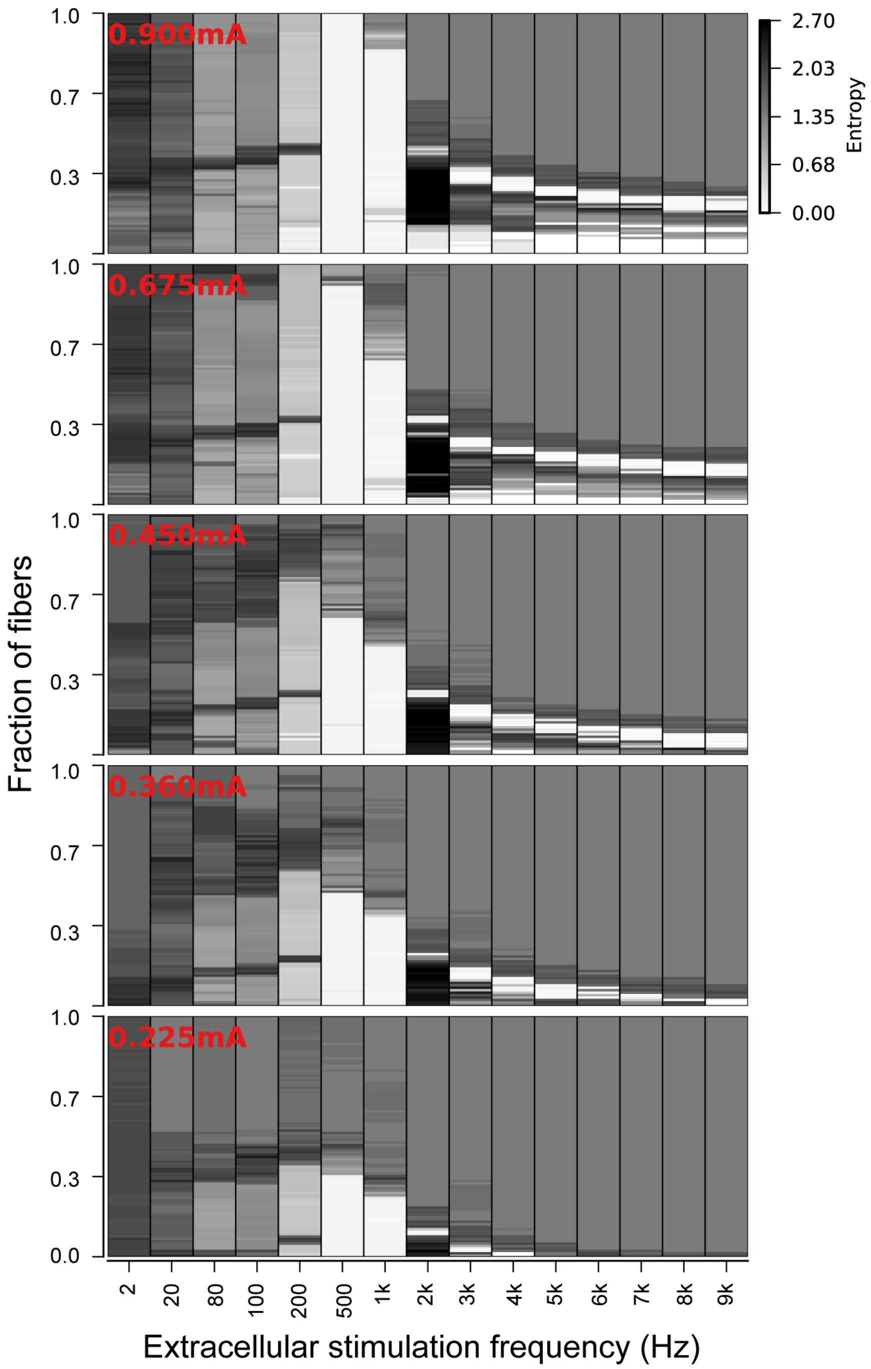

Supplementary Figure 6. Summary entropy colormaps for populations of 5.7µm diameter fibers (100 axons with 81 nodes of Ranvier and intrinsic activity) stimulated with sinusoidal waveforms of increasing amplitudes. Note that although conduction block arises (white bands for frequencies above 1 kHz), the frequency-block threshold relation is inverted in relation to stimulation with cathodic monophasic pulses.

1.2 Supplementary Tables

<table>
<tr><th>Parameter</th><th>Units</th><th>Node</th><th>MYSA</th><th>FLUT</th><th>STIN</th></tr>
<tr><td>Fiber diameter – D</td><td>μm</td><td colspan="4">5.7</td></tr>
<tr><td>Diameter – d</td><td>μm</td><td colspan="2">1.9</td><td colspan="2">3.4</td></tr>
<tr><td>Individual segment length</td><td>μm</td><td>1</td><td>3</td><td>35</td><td>70.5</td></tr>
<tr><td>Number of segments in continuous section</td><td>-</td><td>-</td><td>-</td><td>-</td><td>6</td></tr>
<tr><td>Periaxonal space width</td><td>μm</td><td>-</td><td>0.002</td><td colspan="2">0.004</td></tr>
<tr><td>Number of myelin lamella – nl</td><td>-</td><td>-</td><td colspan="3">80</td></tr>
<tr><td>Membrane capacitance – C_n,C_i</td><td>μF/cm^2/lamella</td><td>2</td><td>0.67</td><td colspan="2">1.19</td></tr>
<tr><td>Myelin capacitance - C_m</td><td>μF/cm^2/lamella</td><td>-</td><td colspan="3">0.1</td></tr>
<tr><td>Myelin conductance - g_m</td><td>S/cm^2</td><td>-</td><td colspan="3">0.001</td></tr>
<tr><td>Intraaxonal resistivity - ρ_a</td><td>Ωcm</td><td colspan="4">70</td></tr>
<tr><td>Periaxonal resistivity – ρ_{pa}</td><td>Ωcm</td><td>-</td><td colspan="3">70</td></tr>
<tr><td>Passive membrane conductances – ρ_{Lk},g_i</td><td>S/cm^2</td><td>0.007</td><td>0.001</td><td colspan="2">0.0001</td></tr>
<tr><td>Maximum fast Na^+ conductance – g_{Na+}</td><td>S/cm^2</td><td>3.0</td><td>-</td><td>-</td><td>-</td></tr>
<tr><td>Maximum fast K^+ conductance – g_{K+}</td><td>S/cm^2</td><td>0.08</td><td>-</td><td>-</td><td>-</td></tr>
<tr><td>Maximum persistent Na^+ conductance – g_{Nap}</td><td>S/cm^2</td><td>0.01</td><td>-</td><td>-</td><td>-</td></tr>
<tr><td>Na^+ Nernst potential – E_{Na+}</td><td>mV</td><td>50</td><td>-</td><td>-</td><td>-</td></tr>
<tr><td>K^+ Nernst potential – E_{K+}</td><td>mV</td><td>-90</td><td>-</td><td>-</td><td>-</td></tr>
<tr><td>Leakage reversal potential – E_{Lk}, E_i</td><td>mV</td><td>-90</td><td colspan="3">-80</td></tr>
<tr><td>Rest potential – E_{rest}</td><td>mV</td><td colspan="4">-80</td></tr>
</table>

Supplementary Table 1. Model details. The inter-nodal segment – STIN – is divided in 6 segments which is represented in NEURON by 6 connected sections with the same parameters and a single segment. The intermodal length is 500μm. The actual myelin sheath capacitance of the model is $C_m/(nl{\times}2)$ and the actual myelin conductance in a similar way.

<table>
<tr><th rowspan="2">Fiber number</th><th colspan="2">Simulation duration</th><th rowspan="2">Stimulation Frequency</th></tr>
<tr><th>1s</th><th>20s</th></tr>
<tr><td>10</td><td>0</td><td>0.05</td><td rowspan="2">200Hz</td></tr>
<tr><td>30</td><td>0.02</td><td>0.05</td></tr>
</table>

40	0	0.05	
50	0.98	1.28	
70	1.34	1.94	
10	1.59	1.62	
30	1.96	2.14	
40	2.09	2.12	2000Hz
50	1.74	1.91	
70	1.42	1.95	
10	0	0	
30	1.2	1.03	
40	2.19	2.35	4000Hz
50	2.01	2.17	
70	1.55	1.96	

Supplementary Table 2. Entropy was slightly underestimated (mean absolute error of 0.19 bits/s) when using short simulation times.
